# Pharmacognosy and technology

**DOI:** 10.4103/0973-1296.66925

**Published:** 2010

**Authors:** D.B.A Narayana

**Affiliations:** *Director, Regulatory Affairs-South Asia, Foods, Home and Personal Care, Unilever Research India, 64, Main Road, Whitefield, Bangalore 560-066, India*

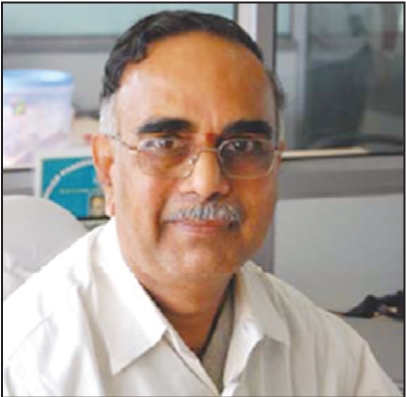



Ever since I joined studies in pharmacy, which is as far as 30 years ago, the subject of pharmacognosy (PC) has been of great interest to me. PC has also seen a lot of ups and downs driven by the growth of pharmaceutical industry, changing consumer habits and desires, and recent consumer movement toward the use of natural materials. There is some resurgence in herbals, which has brought PC again to attention. However, the aspect of identification and study of pharmacognostic properties of raw herbs appears to have not got the attention it deserves. The methodology of teaching students the first few chapters of PC in making them identify morphologic and microscopic characteristics of herbals has not changed much. Though chemotaxonomy, genotaxonomy and some amount of molecular taxonomy have evolved, the routine aspects of methodology used to confirm botanical identity of plants and their parts have remained more or less the same. While the industrial usage of plants and their parts, either as such or after processing, has gone up (with their use in dietary supplements, additives to foods, as ingredients in cosmetics as well as to formulate traditional medicines on industrial scale), the technology for confirming their botanical identity has not seen much changes. Concurrently, the fear of substitution and adulteration of raw herbs is also on the rise. Availability of qualified and experienced botanists and taxonomists, who have expertise in identifying the consignment of plant parts that are coming to an industry, is on the decline as this career is all not very encouraging. Performing macroscopy and microscopy as a routine test and documenting their results properly to meet GMP requirements, especially when large consignments of plant parts are received by industry, is a difficult, time consuming job, and one does not see good documentation leaving doubts about such quality control tests being done. Some of the traditional medicine manufacturers have experienced Ayurvedic Vaidyas (a qualified Ayurvedic practitioner), who are endowed with qualities to look at, taste and evaluate to confirm the herbs they are using to make the medicines, but it is becoming difficult to see such Vaidyas in large numbers. To add to this, controversial botany is another aspect (*Phcog Rev. Vol, 3, Issue 5, 1-7, 2009*). The movement of the industry to use pre-powdered plant parts as a means of reducing processing in the factories and value addition to collectors and cultivators of plant supplies demands identity testing and confirmation from differing substrates of the materials. Newer techniques and technologies need to be evaluated by pharmacognosists (PCs) for this purpose.

All of this will have an impact on the safety, quality and efficacy of the finished product containing a plant or its parts or processed plant materials. Proper identification of the plant used is the first step to build quality and safety. Some of the pharmacopeias have introduced mandatory thin layer chromatography (TLC) testing of the plant material under examination, for their TLC profiles, in comparison with the TLC profile of a botanical reference substance issued by the pharmacopeia commissions. This has improved the identity testing, but cannot replace the botanical identity testing (*Indian Pharmacopoeia, Indian Pharmacopoeia Commission, Min. of Health & FW, Govt. of India, 2007, Appendix 3.2, p 423-439*).

It is also true that one meets PC experts who can reel off individual, distinct, and differentiating characteristics that can confirm identities and also differentiate the substituents and adulterants. Very few books are written on them and even if they are available they suffer from the lack of length (coverage of large number of plants), breadth/depth (coverage of the plant with substituents’ and adulterants’ characteristics). An analysis of the area reveals that there are a number of parameters that can be used in the identification process such as starch grains and their structures, lignified parenchyma, cork and their structures, calcium oxalate crystals, stomata and differences and stomatal index, the most famous trichomes, vessels and tracheids, stone cells, type and varieties of fibers pollen grains, and others.

It is the need of the hour that PCs should consider using today’s technology to aid in proper identification of plant parts, even in their powdered forms. The use of a computer-based system that can identify, count and statistically analyze the number of chromatoids and those chromosomes that have been “aberrated” when one performs a “chromosomal aberration test” as per OECD guidelines is well known. If it can do this, it is not impossible to adopt a similar approach to the powder identification of plant-based materials. Such a system has been well described in the chapter “Microcomputer as an aid in Drug Microscopy” for over 100 plants of western origin. (*Trease and Evans, Pharmacognosy, 13th edition, ELBS, Chapter 43, p 784-797*)

It is not easily understood why Indian PCs have not looked at adopting technology and come up with databank and a computer-based approach for providing technology that can be as easy as “someone taking an IR of a chemical and the PC (personal computer) searching its databases and in less than a minute gives the identity”. A coordinated project with many pharmacy colleges, supported by botanists and software experts can certainly deliver the result. The results of such a project are enormous and do not need to be elaborated or listed here. To begin with, if one attempts to cover about 150 most commonly used medicinal plants of India, along with their substitutes and adulterants (total no. of samples to be analyzed could well be about a maximum of 1000 samples), it would be a great contribution to the science of PC using modern information technology. Funds for such projects are sure to flow from agencies like National Medicinal Plants Board of Ministry of Health (NMPB), Govt. of India, to name one. Adopting such technology does not take away the need for scientists; but the scientists will be using technology.

When will Pharmacognosists (PCs) to talk to personal computers (PCs)?

